# The genomic distribution of intraspecific and interspecific sequence divergence of human segmental duplications relative to human/chimpanzee chromosomal rearrangements

**DOI:** 10.1186/1471-2164-9-384

**Published:** 2008-08-12

**Authors:** Tomàs Marques-Bonet, Ze Cheng, Xinwei She, Evan E Eichler, Arcadi Navarro

**Affiliations:** 1 Unitat de Biologia Evolutiva Departament de Ciències Experimentals i de la Salut, Universitat Pompeu Fabra, Barcelona, Catalonia, Spain; 2 Department of Genome Sciences, University of Washington School of Medicine, Seattle, Washington 98195, USA; 3 Institucio Catalana de Recerca i Estudis Avancats (ICREA) and Universitat Pompeu Fabra, Barcelona, Catalonia, Spain; 4 Population Genomics Node (GNV8), National Institute for Bioinformatics (INB) Universitat Pompeu Fabra, Spain

## Abstract

**Background:**

It has been suggested that chromosomal rearrangements harbor the molecular footprint of the biological phenomena which they induce, in the form, for instance, of changes in the sequence divergence rates of linked genes. So far, all the studies of these potential associations have focused on the relationship between structural changes and the rates of evolution of single-copy DNA and have tried to exclude segmental duplications (SDs). This is paradoxical, since SDs are one of the primary forces driving the evolution of structure and function in our genomes and have been linked not only with novel genes acquiring new functions, but also with overall higher DNA sequence divergence and major chromosomal rearrangements.

**Results:**

Here we take the opposite view and focus on SDs. We analyze several of the features of SDs, including the rates of intraspecific divergence between paralogous copies of human SDs and of interspecific divergence between human SDs and chimpanzee DNA. We study how divergence measures relate to chromosomal rearrangements, while considering other factors that affect evolutionary rates in single copy DNA.

**Conclusion:**

We find that interspecific SD divergence behaves similarly to divergence of single-copy DNA. In contrast, old and recent paralogous copies of SDs do present different patterns of intraspecific divergence. Also, we show that some relatively recent SDs accumulate in regions that carry inversions in sister lineages.

## Background

Initial analyses of the human genome sequence have showed that ~5% of the human genome is composed by interspersed segmental duplications (SDs) [1]. SDs can be defined as blocks of DNA ranging from 1–400 kb in length, with copies found in multiple sites and that typically share high sequence similarity (> 90%). The distribution of these duplications is non-uniform within and among chromosomes, with a tendency to cluster in pericentromeric and subtelomeric regions [2-7] and in the breakpoints of chromosomal rearrangements [8-12]

Duplications have both functional and structural effects [1,2,6,7,9,13-15]. Their functional consequences are very diverse. First, by predisposing chromosomal architectures to be rearranged by non-allelic homologous recombination [7,12,16-18], SDs constitute genetic risk factors for many diseases (e.g. Prader-Willi, Williams-Beuren Syndromes, juvenile nephronophtisis or spinal muscular atrophy). Second, SDs are related to genic evolution because they produce duplications of coding sequences that can lead to genes with new functions [7,19-25]. Finally, rates of evolution of duplicated genes are accelerated just after the duplication event [26]. These accelerations could be due to an increase of mutation rates after duplication, the relaxation of purifying selection due to the duplication of functional genes, the action of positive diversifying selection on one or both copies, or a combination of these factors [5,25-28].

Regarding structural effects, SDs predispose chromosomes to rearrangements, which suggests that SDs may be the main force driving the evolution of genomic structure along the lineages of mammalian species [8-12]. Other studies, however, point to both SDs and chromosomal rearrangements as different manifestations of the intrinsic instability of some particular DNA sequences [9,13,29,30].

Recently, interest in the role of chromosomal rearrangements in speciation processes has been renewed. Models of chromosomal speciation based on the reduction of recombination induced by rearrangements pose that regions involved in those rearrangements could become isolated earlier when compared to the rest of the genome [31-34]. These models predict an association between rearranged regions involved in any speciation process and higher divergence rates of linked DNA sequences. Current evidence for or against such models is extremely contradictory. In human-chimpanzee comparisons, higher evolutionary rates were originally linked to chromosomal rearrangements [35-37], whereas other studies found no effect [38,39] and even more recent ones have detected lower evolutionary rates within inversions [40]. In other lineages, new studies remain consistent with the original finding of higher evolutionary rates associated with chromosomal rearrangements [41-43].

Other explanations have been proposed to account for the relationship between chromosomal rearrangements and faster or slower evolutionary rates. For example, chromosomal rearrangements can influence DNA divergence rates simply by inducing changes in genomic contexts. For instance, if some DNA fragments are moved by a chromosomal inversion from a region with different recombination rates or different equilibrium nucleotide composition, this could induce changes in mutation [44,45]. Also, rearrangements may tend to occur or to be fixed in regions of relaxed purifying selection and, thus, of faster genic evolution [5,36]. Finally, chromosomal rearrangements (especially chromosomal fissions) have been found to be located in regions of ancestrally high GC content in mammals (at least in the Dog genome) [46]. Thus, ancestral GC content could be contributing to the observed relationship between chromosomal rearrangements and higher mutation rates by means of methylation and deamination of CpG dinucleotides, leading to higher divergence measures in regions close (and within) the rearrangements.

Regardless of how the relationship between sequence evolution and chromosomal location change is ultimately resolved, it is important to consider the possibility of an association between SDs and chromosomal rearrangements in relation to speciation. If rearranged chromosomes, whose breakpoints are enriched with SDs, take part in speciation processes in which individuals bearing different chromosomal structures become genetically isolated, it is possible that evolutionary novelties contained in these duplications play some role in such isolation processes.

To tackle this issue we must start by understanding the rates and patterns of SD divergence in the primate lineages. Here, we analyze the genomic distribution of intraspecific divergence between paralogous copies of human SDs and of interspecific divergence between regions duplicated either in humans or chimpanzees and their homologous sequences in the other species. We take into account all major chromosomal rearrangements (see Methods), and, in addition, several other genomic variables that affect evolutionary rates of single copy DNA, such as, linkage to the X chromosome, HSAX [41,47,48], or to telomeric and centromeric regions [40,49-51].

## Results

We addressed three main sets of questions. First, how are SDs distributed in the genome relative to rearrangements? Second, what is the genomic distribution of divergence between paralogous copies of human SDs, especially in relation to rearrangements? And, third, what are the divergence distribution patterns of copies of SDs between humans and chimpanzees? To address each of these questions we used three different datasets (see Material & Methods for a detailed description). The first one (*Raw dataset*) contains pairs of coordinates of fragments of the human genome that have been defined as segmental duplications [1] together with measures of divergence between these paralogous fragments. This dataset is used to detect accumulations of SDs in different parts of the genome. The second dataset (*Non-overlapping intraspecific dataset*) was created to remove redundant information from the previous dataset. It contains only a sample of SDs representative of each duplicated region. Finally, a third dataset (*Non-overlapping interspecific dataset*) was designed to represent the inter-specific divergence between human and chimpanzee for non-overlapping duplicated regions of the human genome. The aim of the two Non-overlapping datasets is to study the distribution of SD divergence rates in different regions of the genome while avoiding the redundant information that the first dataset contains. To do so, the simplifying assumption is made that the selected representative of each duplicated region actually reflects the complex history of the region.

### Overrepresentation of relatively young SDs in rearranged regions

We started using the raw dataset (Dataset 1, see Methods) to study the distribution of paralogous copies of human SDs relative to the nine major rearrangements (Inversions) between humans and chimpanzees (human chromosomes 4, 5, 9, 12, 15, 16, 17 and 18). We defined as "young" SDs those with a greater than 98% sequence identity among copies, while SDs with less than 92% identity were labeled as "old". These labels, of course, do not imply strict age estimates, since gene conversion or positive selection are known to influence divergence rates of SDs.

**Figure 1:**
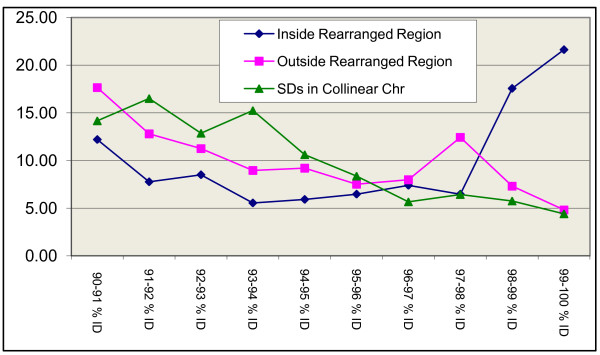
**Distribution of SDs identities relative to major rearrangements (Inversions) between humans and chimpanzees.** In Blue, the distribution of percentages of SDs that are located within the inversion of human chromosomes rearranged relative to chimpanzees. In pink, the distribution of SDs in rearranged chromosomes but outside the rearrangements. In green the percentages of identities of SDs located in chromosomes that are collinear (not rearranged) for both species.

After all the filtering processes (see Methods) in the filtered dataset, we observed a higher proportion of young SDs within rearranged regions than outside them: ~40% of SDs located within rearranged regions are young, while this figure is only ~12% for SDs outside the inverted regions of the same chromosomes. Also, these regions contained younger SDs than colinear chromosomes, where only ~11% of SDs are classified as young (Table 1, Figure 1). It is crucial to note that these young duplications cannot be caused by the inversions. Most of the 10 major rearrangements separating humans and chimpanzees took place in the chimpanzee lineage [52], and here we are analyzing human SDs. Thus, this association is not caused by an accumulation of SDs within the inversion itself, but within the orthologous region in the homologous chromosome of the sister species, which retained the ancestral structure.

**Table 1:** Distribution of SD identities relative to major genomic rearrangements between humans and chimpanzees.

	**Inside rearranged regions**	**Outside rearranged regions**	**Colinear Chromosome**
Similarity(ID)	Percentage of age within each cathegory (%)		

90–91% ID	12.20	17.64	14.16
91–92% ID	7.76	12.79	16.50
92–93% ID	8.50	11.25	12.86
93–94% ID	5.55	8.96	15.24
94–95% ID	5.91	9.19	10.61
95–96% ID	6.47	7.51	8.36
96–97% ID	7.39	7.98	5.67
97–98% ID	6.47	12.42	6.43
98–99% ID	17.56	7.31	5.76
99–100% ID	21.63	4.81	4.41

The percentages were calculated as the proportion of pairwise alignments at each percent identity.

**Figure 2:**
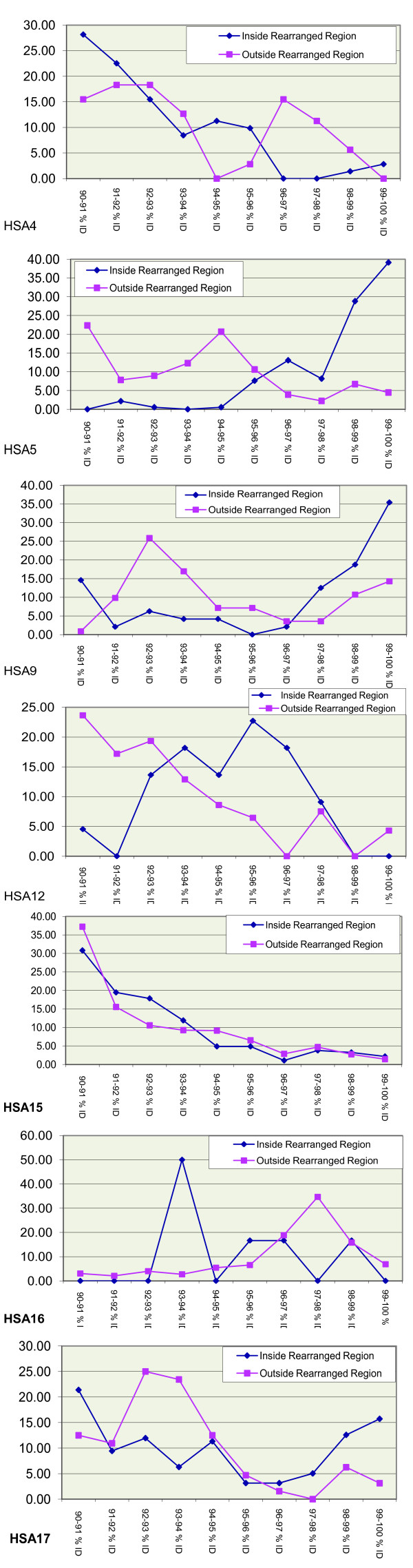
**Distribution of identities relative to major rearrangements between human and chimpanzees for individual chromosomes.** Chromosomes without any pair of copies of SDs within rearrangements are not shown (see Methods).

To check whether these results were due to a genome-wide phenomenon or were driven by some individual chromosomes, we performed a chromosome by chromosome analysis. This allowed us to pinpoint HSA5 and HSA9 as primarily responsible for the reported association. These chromosomes show the largest difference in percent identity and correspond to the greatest proportion of alignments (total number of SD pairs). No other chromosome showed a differential accumulation of young SDs within their rearrangements (Figure 2). Therefore, the association above is mainly due to these two chromosomes which, being inverted in one lineage (chimpanzee), have accumulated an expansion of recent SDs in its sister lineage (human).

**Figure 3:**
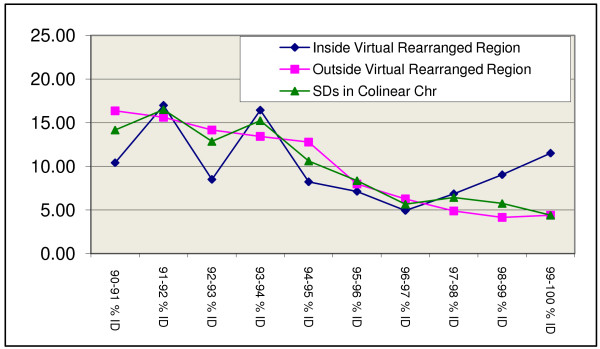
Distribution of SDs identities relative to simulated pericentromeric rearrangements in colinear chromosomes between humans and chimpanzees.

**Figure 4:**
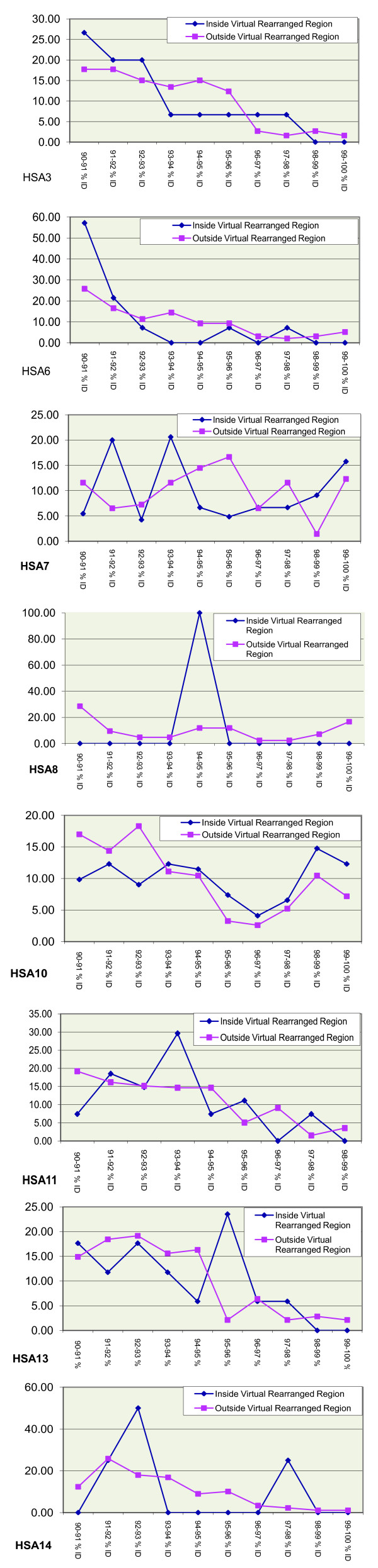
Distribution of SDs identities relative to simulated pericentric rearrangements in colinear chromosomes between humans and chimpanzees for individual chromosome. Chromosomes without any pair of copies of SDs within simulated rearrangements are not shown (see Methods).

Given that SDs tend to cluster within pericentromeric and subtelomeric zones [1,5,50], part of the above effect could be attributed to the fact that all the major rearrangements between humans and chimpanzees are pericentric, and thus include the centromere. We accounted for this possibility by excluding SDs that mapped within 5 Mb of the centromeres. To make sure that the filtering process had eliminated any centromere-associated effect, we simulated pericentric inversions in colinear chromosomes and searched for young SDs within them. Pseudo-inverted pericentric regions in colinear chromosomes were defined as regions equivalent in length and location to real rearrangements. Given that the average inversion spans 24.98% of its chromosome, we created a virtual inversion of that size in each colinear chromosome, keeping the centromere as the center of the inversion. On average, chromosomes with virtual inversions did present a higher proportion of young SDs, but the effect is not as large. First, the increase was only 50% of that in real inversions (Table 2, Figure 3); and second, only HSA10 and HSA7 seemed to accumulate some local clustering of recent SDs (Figure 4). However, clustering is not exclusive of the inverted region, as is the case for the inverted chromosomes HSA5 and HSA9, but extends all over the chromosome. The rest of the colinear chromosomes did not show any particular age distribution of SDs inside *vs*. outside virtual rearrangements, suggesting that the association of young SDs and rearranged chromosomes 5 and 9 might be not only due to the accumulation of SDs near centromeres, even if that accumulation is likely to make a major contribution to the magnitude of our observation.

**Table 2:** Distribution of SDs identities relative to simulated rearrangements in colinear chromosomes between human and chimpanzees.

	**Inside rearranged regions**	**Outside rearranged regions**
Similarity(ID)	Percentage of age within each category (%)	

90–91% ID	10.41	16.35
91–92% ID	16.99	15.62
92–93% ID	8.49	14.16
93–94% ID	16.44	13.43
94–95% ID	8.22	12.77
95–96% ID	7.12	7.97
96–97% ID	4.93	6.27
97–98% ID	6.85	4.88
98–99% ID	9.04	4.15
99–100% ID	11.51	4.39

The percentages were calculated as the proportion of pairwise alignments at each percent identity.

### The distribution of divergence between human paralogous SDs

To study how the rates of intraspecific evolution of SD may be affected by rearrangements and other factors such as the location in sex chromosomes or telomeres, we used a second dataset: the non-overlapping dataset or Dataset 2 (see Methods). Given the results above, we extracted two subsets from the original dataset: "young SDs" (> 98% ID) and "old SDs" (< 92% ID). We kept, as representatives of every covered zone, SDs that had both copies in the same class of region (see Additional file 1).

We sequentially analyzed and removed every known variable affecting divergence rates (Table 3), starting with sex chromosomes. Young human SDs located in HSAX presented less divergence among copies than equivalent SDs in autosomes. This is not the case for old SDs. No length differences were detected in SDs located in HSAX. When located in HSAY, young SDs presented lower intra-specific divergence and increased length. Old SDs in HSAY are also longer, but, in contrast, they present higher divergence between paralogous copies.

**Table 3:** Average of divergences and lengths among paralogous copies of SDs relative to genomic factors and rearrangements between human and chimpanzees.

Sex Chromosomes				ID
	**SDs in Autosomes**	**SDs in HSAX**	**P-value**	**> 98%**

**N**	889	103		
**K**	0.0107	0.0076	< 0.001	
**Size**	41,439.50	52,887.93	0.115	

	**SDs in Autosomes**	**SDs in HSAX**	**P-value**	**< 92%**

**N**	3273	261		
**K**	0.0958	0.0962	0.364	
**Size**	4,689.73	4,458.48	0.578	

	**SDs in Autosomes**	**SDs in HSAY**	**P value**	**> 98%**

**N**	889	32		
**K**	0.0107	0.0052	< 0.001	
**Size**	41,439.50	########	< 0.001	

	**SDs in Autosomes**	**SDs in HSAY**	**P value**	**< 92%**

**N**	3273	132		
**K**	0.0958	0.0976	0.001	
**Size**	4,689.73	12,290.17	< 0.001	

Telomeres (10 Mb)				ID

	**SDs not in telomeres**	**SDs in Telomeres**	**P-value**	**> 98%**

**N**	719	170		
**K**	0.0105	0.0115	0.052	
**Size**	44,040.90	30,437.11	0.01	

	**SDs not in telomeres**	**SDs in Telomeres**	**P-value**	**< 92%**

**N**	2831	442		
**K**	0.0958	0.0958	0.874	
**Size**	4,746.56	4,325.72	0.224	

Centromere (5 Mb)				ID

	**SDs not in Centromeres**	**SDs in Centromeres**	**P-value**	**> 98%**

**N**	572	147		
**K**	0.0106	0.01	0.316	
**Size**	36,111.33	74,896.07	< 0.001	

	**SDs not in Centromeres**	**SDs in Centromeres**	**P-value**	**< 92%**

**N**	2096	735		
**K**	0.0959	0.0953	0.029	
**Size**	3,908.13	7,137.51	< 0.001	

HSA19				ID

	**SDs in other autosomes**	**SDs in HSA19**	**P-value**	**> 98%**

**N**	561	11		
**K**	0.0105	0.0126	0.32	
**Size**	36,600.01	11,188.90	0.139	

	**SDs in other autosomes**	**SDs in HSA19**	**P-value**	**< 92%**

**N**	2029	67		
**K**	0.0959	0.0958	0.906	
**Size**	3,875.29	4,902.67	0.141	

Rearranged Chromosomes				ID

	**SDs in Colinear chr**	**SDs in Rearranged Chr**	**P-value**	**> 98%**

**N**	208	353		
**K**	0.0114	0.01	0.009	
**Size**	25,385.48	43,208.01	< 0.001	

	**SDs in Colinear chr**	**SDs in Rearranged Chr**	**P-value**	**< 92%**

**N**	890	1139		
**K**	0.0959	0.096	0.902	
**Size**	3,791.72	3,940.59	0.534	

Inside rearranged regions versus Outside rearranged regions, without HSA2				ID

	**SDs Outside rearranged regions**	**SDs Inside rearranged regions**	**P-value**	**> 98%**

**N**	216	87		
**K**	0.0104	0.0096	0.347	
**Size**	40,400.12	55,156.56	0.058	

	**SDs Outside rearranged regions**	**SDs Inside rearranged regions**	**P-value**	**< 92%**

**N**	715	267		
**K**	0.096	0.0957	0.586	
**Size**	3,879.46	4,868.64	0.016	

Inversions detected in (Newman et al 2005) vs rest of chromosomes				ID

	**SDs Outside Inversion**	**SDs Inside Inversion**	**P-value**	**> 98%**

**N**	541	20		
**K**	0.0104	0.0131	0.063	
**Size**	35,170.62	75,264.90	0.003	

	**SDs Outside Inversion**	**SDs Inside Inversion**	**P-value**	**< 92%**

**N**	1977	52		
**K**	0.0959	0.0986	0.004	
**Size**	3,853.94	4,686.98	0.281	

Breakpoints versus inverted chromosomes (excluding HSA2)				ID

	**SDs rest of Chr**	**SDs in BKP**	**P-value**	**> 98%**

**N**	286	17		
**K**	0.0103	0.008	0.135	
**Size**	44,189.30	52,171.11	0.611	

	**SDs rest of Chr**	**SDs in BKP**	**P-value**	**< 92%**

**N**	953	29		
**K**	0.096	0.0941	0.15	
**Size**	4,159.23	3,792.82	0.748	

Divergence (K) is calculated as the number of substitution per site between the two duplication alignments. Length (Size) corresponds to the aligned basepairs. P-values are calculated by means of permutation test (see Material and Methods).

Regarding the position of SDs along chromosomes, we first considered telomeres. Only young SDs located in telomeres showed higher divergence between paralogous copies. They also showed shorter alignment sizes. On the contrary, old SDs did not present divergence differences between telomeres and the rest of the genome. When focusing on centromeres, we found that SDs near them are longer in both subsets (young and old SDs). As to divergence, only old SDs showed a slight decrease of paralogous divergence in pericentromeric regions compared to SDs located elsewhere in the genome.

HSA19 has been shown to have atypical divergence and nucleotide composition patterns. It presents higher divergence between human and mice, higher GC content, and an accumulation of DNA binding genes [53,54]. Also, HSA19 appears to have a deficit of interspersed SDs (as opposed to tandem) [5,53]. Surprisingly, our analysis shows that SDs located in this chromosome did not differ from SDs in other autosomes, neither in their length nor their divergence rates.

When we finally compared paralogous copies of human SDs located in rearranged chromosomes *versus *SDs located in colinear chromosomes, the only detectable patterns were that young SDs are significantly longer and less divergent when located in rearranged chromosomes. However, this observation can not be exclusively attributed to inversions; because when comparing divergence among copies of human SDs within the inverted regions (recall that most rearrangements took place in the chimpanzee lineage) *versus *SDs outside the inversion in rearranged chromosomes, there were no divergence differences, although SDs were longer within rearranged regions. Since evolutionary breakpoints are enriched with SDs in many species [8-12], we assessed the sequence features of SDs located at the breakpoints of inversions separating humans and chimpanzees. Neither the length nor the divergences of those SDs are statistically different from SDs located elsewhere in the genome.

Finally, we considered a set of small inversions recently detected *in silico *[55]. SDs located within these inversions showed a slight increase in divergence (highly significant for old SDs and marginally significant for young SDs). Only young SDs showed a remarkable increase of length within those rearrangements.

### The distribution of divergence between human and chimpanzee SDs

We used Dataset 3 (*Non-overlapping interspecific dataset*, see Methods) to study divergence between human and chimpanzee SDs. This dataset is formed by two subsets of SDs: first, a subset non-overlapping human SDs for which we have measures of divergence from chimpanzee; and second, a subset of non-overlapping chimpanzee SDs for which we have measures of divergence from human (see Additional file 2). Again, we studied the effect of all the factors considered above in the divergence of SDs among species by sequentially analyzing and removing every individual factor (Table 4).

**Table 4:** Average of inter-specific divergences in human SDs and chimpanzee SDs relative to genomic factors and rearrangements between human and chimpanzees.

**HUMAN SD**	**X-Chromosome**		**Y-Chromosome**		**Telomeres vs. rest of genome**		**Centromeres vs. rest of genome**		**Chromosome 19**	
								
	**vs. Autosomes**		**vs. Autosomes**							
	**SDs in autosomes**	**SDs in the HSAX.**	**SDs in autosomes**	**SDs in the HSAY.**	**SDs outside Telomeres**	**SDs within Telomeres**	**SDs outside Centromeres**	**SDs within Centromeres**	**SDs outside HSA19**	**SDs within HSA19**

***N***	*1303*	*109*	*1303*	*51*	*1052*	*251*	*742*	*310*	*714*	*28*
**Divergence**	0.0238	0.0161	0.0238	0.0259	0.0233	0.026	0.0228	0.0247	0.0225	0.0285
**P-value**		< 0.001		0.087		< 0.001		< 0.001		0.001

**CHIMP SD**	**X-Chromosome**		**Y-Chromosome**		**Telomeres vs. rest of genome**		**Centromeres vs. rest of genome**		**Chromosome 19**	
								
	**vs. Autosomes**		**vs. Autosomes**							

	**SDs in autosomes**	**SDs in the HSAX.**	**SDs in autosomes**	**SDs in the HSAY.**	**SDs outside Telomeres**	**SDs within Telomeres**	**SDs outside Centromeres**	**SDs within Centromeres**	**SDs outside HSA19**	**SDs within HSA19**

***N***	*1415*	*110*	*1415*	*87*	*1224*	*191*	*789*	*435*	*779*	*10*
**Divergence**	0.0222	0.0156	0.0222	0.0223	0.0217	0.0252	0.021	0.0231	0.0207	0.038
**P-value**		< 0.001		0.891		< 0.001		< 0.001		< 0.001

**HUMAN SD**	**Rearranged vs.**			**SDs within vs.**						
			
	**Colinear chromosomes**			**outside rearranged regions (excluding HSA2)**						

	**SDs in colinear chr.**	**SDs in Rearranged chr.**	**P-value**	**SDs Outside inversions**	**SDs inside inversions**	**P-value**				

***N***	*267*	*447*		*280*	*112*					
**Divergence**	0.0236	0.0219	0.01	0.0218	0.0219	0.934				

**CHIMP SD**	**Rearranged vs.**			**SDs within vs.**						
			
	**Colinear chromosomes**			**outside rearranged regions (excluding HSA2)**						

	**SDs in colinear chr.**	**SDs in Rearranged chr.**	**P-value**	**SDs Outside inversions**	**SDs inside inversions**	**P-value**				

***N***	*256*	*523*		*312*	*160*					
**Divergence**	0.0216	0.0203	0.025	0.0202	0.0199	0.693				

**HUMAN SD**	**Breakpoints vs.**									
		
	**inverted chromosomes**									
		
	**(excludingHSA2)**									

	**SDs in Rearranged chr.**	**SDs in BKPs**	**P-value**							

***N***	*370*	*22*								
**Divergence**	0.0217	0.0242	0.136							

**CHIMP SD**	**Breakpoints vs.**									
		
	**inverted chromosomes**									
		
	**(excludingHSA2)**									

	**SDs in Rearranged chr.**	**SDs in BKPs**	**P-value**							

***N***	*441*	*31*								
**Divergence**	0.0201	0.0194	0.552							

**HUMAN SD**	**SDs within vs. outside rearranged regions**									
		
	**(excluding breakpoints and HSA2)**									

	**SDs Outside inversions**	**SDs inside inversions**	**P-value**							

***N***	*264*	*106*								
**Divergence**	0.0216	0.022	0.619							

**CHIMP SD**	**SDs within vs. outside rearranged regions**									
		
	**(excluding breakpoints and HSA2)**									

	**SDs Outside inversions**	**SDs inside inversions**	**P-value**							

***N***	*291*	*150*								
**Divergence**	0.0202	0.0199	0.583							

**HUMAN SD**	**inversions (Newman et al. 2005) versus rest chromosomes**									
		
	**SDs Outside inversions**	**SDs inside inversions**	**P-value**							

***N***	*670*	*44*								
**Divergence**	0.0227	0.0196	0.015							

**CHIMP SD**	**inversions (Newman et al. 2005) chromosomes**									
		
	**SDs Outside inversions**	**SDs inside inversions**	**P-value**							

***N***	*713*	*66*								
**Divergence**	0.021	0.0183	0.004							

Divergence is calculated as the number of substitution per site between the two duplication alignments.

Our first observation was that SDs located in HSAX showed lower divergence than SDs in autosomes. This effect was consistent for both datasets of inter-specific SD divergence. Second, regions near telomeres presented higher divergence than the rest of the chromosome, just as previously seen for single-copy DNA in other studies [51,40]. This pattern was again consistent for both human and chimpanzee subsets of SDs. In contrast, and contrary to other studies [40,41], inter-specific divergence in SDs is higher near pericentromeric regions. Finally, SDs in HSA19 present higher divergence than SDs in other autosomes (Table 4).

Regarding the effect of rearrangements over interspecific SD divergence, we found that SDs within rearranged chromosomes diverged less than SDs in colinear chromosomes, which is in agreement with the most recent results for single copy genes [40]. In contrast to previous results, there were no significant divergence differences between SDs within *versus *SDs outside rearranged regions. Finally, and again differing from results in single copy genes [40], SDs located within small inversions [55] revealed lower divergence rates compared to SDs located elsewhere in the genome. To unveil any specific individual contributions of chromosomes, we analyzed interspecific divergence for every inversion (Table 5). There was no clear pattern to be detected. Only HSA9 presented higher divergence within its inversion and only when considering the subset of chimpanzee SDs.

**Table 5:** Average of inter-divergences in human SDs and chimpanzee SDs in individual chromosomes relative to major rearrangements between human and chimpanzee.

Hs Chr	**Human SDs**					**Chimp SDs**					
			
	**Outside rearranged regions**	**Inside rearranged regions**	P-value	*N*_*out*_	*N*_*in*_	**Outside rearranged regions**	**Inside rearranged regions**	P-value	*N*_*out*_	*N*_*in*_	Lineage of the rearrangement
HSA1	0.0209	0.0070	0.043	*106*	*1*	0.0203			*105*	*0*	HUMAN
HSA4	0.0246	0.0263	0.608	*17*	*13*	0.0247			*13*	*0*	CHIMP
HSA5	0.0226	0.0170	0.116	*10*	*16*	0.0197	0.0161	0.065	*10*	*57*	CHIMP
HSA9	0.0230	0.0246	0.440	*35*	*26*	0.0184	0.0232	< 0.001	*49*	*38*	CHIMP
HSA12	0.0201	0.0243	0.286	*9*	*7*	0.0216	0.0190	0.875	*1*	*8*	CHIMP
HSA15	0.0251	0.0239	0.661	*41*	*7*	0.0224	0.0239	0.418	*48*	*10*	CHIMP
HSA16	0.0188	0.0319	0.008	*41*	*2*	0.0181	0.0413	0.008	*68*	*1*	CHIMP
HSA17	0.0215	0.0198	0.427	*16*	*40*	0.0225	0.0206	0.352	*17*	*46*	CHIMP
HSA18	0.0245			*5*	*0*	0.0252			*1*	*0*	HUMAN

**TOTAL **(without HSA2)	**0.0218**	**0.0219**	**0.934**	***280***	***112***	**0.0202**	**0.0199**	**0.693**	***312***	***160***	

## Discussion

Several conclusions arise from our whole-genome SDs analysis. First, there is an accumulation of relatively recent human SDs within some chromosomes that carry an evolutionary rearrangement between human and chimpanzees. Seven of the nine major inversions between humans and chimpanzees occurred in the chimpanzee lineage (HSA4, HSA5, HSA9, HSA12, HSA15, HSA16 and HSA17), thus inversions cannot be the cause of that accumulation. The classical explanation of the accumulation would be that some of these young SDs predate the split of humans and chimpanzees and, thus, that they originated the inversions via non-allelic homologous recombination, but this seems unlikely in the light of their location. Our observations are consistent with an alternative scenario in which both chromosomal rearrangements and SDs are consequences of a third factor, perhaps regions of high instability [29,56]. This has been suggested in opposition to the idea that rearrangements and SDs are related only because highly similar regions promote rearrangements by non-allelic recombination [8-12]. A final possibility is that we are observing an excess of similar duplications in pericentromeric regions, specially in HSA5 and HSA9, in which there are an excess of young human SDs (> 98% ID) within regions that were inverted in chimpanzees. Even if we endeavored to remove the effect of centromeres, the possibility remains that particularly strong local effects were not accounted for. Only further research on primate SDs will allow to ascertain the involved phenomena and the order in which they occurred.

Several authors have found that the association among rearrangement breakpoints and segmental duplications is maintained between different lineages, but not within the same lineage [6,9,13]. For instance, primate segmental duplications occur at specific locations that are enriched for mouse-human synteny and mouse-rat synteny breaks. As the majority of synteny rearrangements have occurred in the rodent lineage, there cannot be a causal relationship between the two. Rather, it must be the case that primate segmental duplications tend to appear at the same locations in which rodent chromosomes have rearranged. Thus, instability would seem a long standing property of these genomes at these locations. In addition, She et al. [5] described a non-uniform distribution of intrachromosomal human SDs and highlighted nine autosomal human chromosomes with an excess of young human SDs, seven of which presented rearrangements between humans and chimpanzees (out of which five were chimpanzee specific). These observations provide evidence for a link between expansions of recent SDs in one lineage and chromosomal rearrangements in the other. Only deeper analysis of the two chimpanzee chromosomes that carry human-specific rearrangements (HSA1 and HSA2) will help to clarify any direct relationship among chromosomal rearrangements and expansion of SDs. This analysis, however, is beyond the scope of the present work and would require a higher quality sequence assembly of the chimpanzee genome.

Several explanations can be put forward as to why chromosomal rearrangements and young SDs should accumulate in sister lineages. The first one relates to the aforementioned instability regions. A recent change in the understanding of the evolution and behavior of SDs [56-58] poses that there are "core elements" that may act as sources for the dispersal of new SDs, by creating a large number of copies of themselves. These copies tend to cluster by means of local duplications. Thus, one explanation for our results would be that some core elements were present in the chromosomes ancestral to those that currently harbor inversions and SDs in humans and chimpanzees. As inversions decrease recombination between homologous chromosomes [31-33], core elements becoming active and expanding by local copies in a given class of chromosome, would be less likely to be eliminated by recombination from their source regions while rearrangements are still segregating in the ancestral population. Thus, these core elements would accumulate copies of themselves only in the lineage in which they appeared. Moreover, the reduction of recombination caused by inversions [59] may also prevent the dispersal of the other associated SDs (not just the "core" elements). SDs trapped within rearrangements would be more similar to the "original" state because they would be prevented from invading other regions or chromosomes that could affect mutation rates and thus produce highly divergent SDs copies.

A second possibility is that lower recombination rates themselves could help explain our results. As suggested in previous work [60-63], there is a positive correlation among low recombination rates, low diversity within species, and low divergence that can be explained by a mutagenic effect of recombination. While inversions are segregating, regions within rearrangements have lower recombination rates and, thus, they should present lower divergence (either inter-specific or intra-specific). Of course, this would only be the case if rearrangements had been segregating in the population for a long time, so that the reduction of recombination could have a detectable impact on mutation rates.

Finally, some of the pairwise alignments classified as young SDs may in fact not be young, but their high identity may have been maintained by gene conversion [6]. Gene conversion is a homogenizing force that might erase differences among copies leading to underestimations of the age of SDs. It is possible that during the segregation of new rearrangements, the resolving structure of the few recombination events taking place within inversions would be biased towards increased gene conversion instead of the reciprocal exchange of chromatids. This would help explain the excess of highly similar tracks of SDs in one lineage together with inversions in the other lineage. However, this possibility implies that most gene conversion events ought to have happened before the separation of the two lineages and while the inversions were segregating in the population, which is unlikely. Moreover, She et al. [5] concluded that gene conversion events can not explain most of the high sequence identity of SD copies.

Secondly, we conclude that old and young SDs evolve at different rates when compared to single-copy DNA, hinting at different evolutionary trajectories for different SD classes. It is possible that young SDs are reflecting the history of recent primate evolution – which led to our species – while old SDs may reflect periods of duplication early during primate evolution. Our results, for example, support a recent expansion of young SDs or a more complex interaction among recombination and SDs. The latter appears to be the case for SDs in telomeres, where young SDs are marginally more divergent, but are significantly shorter than elsewhere in the genome, maybe as a result of telomeres having higher rates of recombination [64,65]. In contrast, older SDs do not show this trend, which could be expected since telomeres are likely to have moved during primate evolution [66,67].

Regarding centromeres, and probably as a result of their decreased recombination rates [64,65], we obtained larger sizes of pairwise alignments of SDs. However, as centromeres have been reported to be prone to repositioning during evolution [68], this result could be reflecting some other cause rather than a direct recombination effect. SDs in HSAY are also longer, which could be related to the lack of recombination in that chromosome or with recent, HSAY-specific, SD expansions.

Our main conclusion regarding major rearrangements between humans and chimpanzees is that young SDs located in rearranged chromosomes are longer and exhibit greater sequence identity than SDs located in colinear chromosomes. This could be expected, since rearrangements are known to be either human or chimpanzee specific and, thus, old SDs should not be affected by such recent rearrangements. Still, both young and old paralogous copies of SDs tend to be larger within rearranged chromosomal regions. This is also the case for smaller rearrangements that have been detected *in silico *[55]. These are puzzling patterns, hinting at some period of decreased recombination within rearranged regions. Finally, we observed higher levels of intraspecific divergence between SDs within smaller inversions [55]. Altogether, these data suggest that chromosomal rearrangements might have affected SD divergence rates during primate evolution.

Our third and last finding is that interspecific SD divergence displays rates and patterns that are roughly equivalent to those of single-copy DNA. SDs located in telomeres and in HSA19 show higher levels of interspecific SD divergence. Also, SDs located in rearranged chromosomes show lower divergence between species. Still, there are some discrepancies between single-copy and duplicated DNA, such as the higher divergence between SDs located in centromeres or the lower divergence of SDs within small inversions. Finally, HSAY does not show the higher degree of divergence reported for single-copy DNA [40-42], perhaps as the result of the recent expansion of young SDs in that chromosome [5] or of extensive gene conversion [69].

As to individual inversions, HSA9 stands out as the only chromosome showing significantly higher human-chimpanzee divergence within its rearrangement. This suggests a burst of interspecific divergence within the inversion, that could perhaps predate speciation. Therefore, HSA9 is currently the best candidate to further study any potential relationship among SDs, rearrangements, divergence, and speciation. If chromosomes have played any role in any of the speciation events that led to humans and chimpanzees, it is clear that not all of them would have made the same contributions and, thus, would not bear the same molecular signatures. We should keep this in mind when trying to explain why HSA4, which presents high divergence of single copy DNA located within its inversion [40], does not present any particular pattern when considering its duplications. Also, certain chromosomes (such as HSA4, HSA5, HSA9, HSA15 and HSA16) have been pinpointed as the most dissimilar between humans and chimpanzees in terms of the expression intensities of their genes [70], findings which are only partially consistent with the results presented here.

## Conclusion

In summary, we conclude that some rearrangements in the human and chimpanzee genome may be associated with dynamic regions in the genome that may result in rearrangements in one lineage and duplications in the other, although the effect is not seen in all chromosomes. On the other hand, intraspecific and interspecific divergences between SDs are affected by the same factors which were known to affect divergence rates of single copy DNA sequences. Although chromosomal rearrangements do affect the evolution and fate of SDs, chromosomal speciation (and its relation with SDs novelties) does not seem to have been a common process along the human and chimpanzee lineages. Still, HSA9 is the best possible candidate to have been involved in some complex interaction among rearrangements, SDs, and evolutionary novelties. Studies which include more species and focus on the powerful novelty-generating force of segmental duplications are needed to increase our knowledge of this exciting topic.

## Methods

### Structural information

Coordinates of telomeres and centromeres of all chromosomes were obtained from Build 35 of the human genome and NCBI Build 1 of the chimpanzee genome [71]. We considered as rearranged chromosomes all those for which major chromosomal rearrangements in either the human or the chimpanzee lineages have been evidenced by recent *in silico *[51,72] or cytological structures [73-77]. This comprised HSA1, HSA4, HSA5, HSA9, HSA12, HSA15, HSA16, HSA17 and HSA18, which differ by a pericentric inversion, and human chromosome 2, which has been generated by an ancestral telomere-telomere fusion [78]. For all chromosomes, all *in silico-*estimated coordinates were compared with newly available cytological data in order to confirm inversion coordinates, as previously done [40]. When indicated, the mini-inversions detected "in silico" by [55] have been used.

### Source of SD data

We retrieved information of segmental duplication about Human and Chimpanzee SDs from the Segmental Duplication Database [79,80]. In brief, we used the whole genome assembly comparison (WGAC), composed by SDs that were detected by the Blast-based method [1] to identify pairwise of DNA sequence of high similarity within the human assembly (Build 35).

Three datasets were built for analysis.

1) Dataset 1. Raw dataset. This is the standard dataset as downloaded from the Segmental Duplication Database. It contains pairs of coordinates of fragments of the human genome that fit two criteria: each pair has a minimum overlap size of 1 kb and presents > 90% identity among copies. [1]. A divergence measure was calculated for every pairwise detection as the number of substitutions per site (applying Jukes-Cantor correction). Besides divergence we also recorded the overlapping size (length) of every pair.

2) Dataset 2. Non-overlapping intraspecific dataset. Because of the methodology used in WGAC, most fragments in the raw dataset are repeated in many partially overlapping pairs, thus adding the same information several times especially in SD clusters. To eliminate this redundant information, we constructed a new dataset containing samples of SDs representative of every region of the genome covered by SD.

The steps used to construct our new dataset were as follows:

2.a. We constructed a "coverage map of SDs". We recorded the bound coordinates of overlapping SDs thus reporting every region in the human genome in which there are SDs. If two coverage zones were separated by a distance lower than 10 kb we joined them to avoid over-representing some parts of the genome. This procedure is similar that the one used in [5], when constructing "duplication hubs", that is, regions with an excess of aligned SDs.

2.b. From this coordinate list and for every "covered" region, we kept only one pair of SD as a representative of the region. The criteria to select one SD against the others were (1) Longer SDs were preferred, as measured by percentage of occupancy within that coverage zone and (2) SDs that had both paralogous copies in the same class of regions. That is, if one coverage zone is in, say, a telomere, we kept the longer SD having its paralogous copy also in a telomere. In case of not having copies in comparable regions, we just keep the longest. We considered seven classes of genomic location: sex chromosomes, telomeres, centromeres, HSA19, colinear chromosomes, colinear regions in rearranged chromosomes, rearranged regions (inversions) and rearrangement breakpoints. The goal of these criteria is to retrieve some non-redundant basic information of this portion of the genome. (see Additional file 1 for a schematic view of the process).

The coverage map to create the non-overlapping datasets was constructed *a posteriori *of the splitting between "young" and "old" duplications. This was done to avoid a bias the selection of a sample SDs for every region. A bias could have been possible since old segmental duplications are shorter than young ones, probably as a result of recombination or subsequent deletion events that breakdown their structure [5]. Thus, if we followed our criteria (for instance the higher coverage criterion (see Methods)) before splitting between young and old SDs, the latter would have had lower probabilities of being selected as a sample of the region of interest

3) Dataset 3. Non-overlapping interspecific dataset. This third dataset was designed to recover a sample of divergence between humans and chimpanzees in regions covered by SDs. From the coverage map of human SD we recovered chimpanzee WGS (v1) sequences [5]. For every "covered" zone (a slice of coordinates), we split it in non overlapping windows of 5000 bp. For every one of those windows, divergence was calculated as the average of all chimpanzee WGS sequences against the human sequence. Finally the average of all windows was computed as the average divergence of the coverage zone. Divergence was calculated applying Kimura's correction. We also constructed a parallel dataset computing divergence of the chimpanzee SDs from human WGS sequences (see Additional file 2).

These three datasets were built in order to tackle different questions. To detect clusters of SDs in some parts of the genome we used the raw dataset, which provides a good perspective of the amount of SDs in every region. When we aim to study divergence in different regions of the genome while avoiding some biases such as overlapping SDs or copies in non-comparable regions of the genome, we should use the non overlapping datasets, either for intraspecific divergence (dataset 2) or interspecific divergence (dataset 3).

### Filtering

Previous to every analysis we performed a sequential filtering process to remove the genomic variables that are known to affect evolutionary rates in single copy DNA. These factors include linkage to sex chromosomes [47,81-83], to telomeres (10 Mb from the tip of chromosome) [51], to centromeres (5 Mb around them) [49,50,84] and to human chromosome 19 (HSA19) [53]. After getting the result for each one of the categories, those SDs located in that specific category were removed from the analysis. As an example, after analyzing the effect of sex chromosomes on our SDs dataset, we removed SDs in sex chromosomes and analyzed the effect of telomeres on the remaining dataset. We also eliminated pairs of SDs that had one copy in rearranged regions and the other copy in colinear regions (since it is impossible to classify that pair as SDs in rearranged or collinear regions).

### Permutation tests

SDs divergence measures in different categories were compared by means of pairwise permutation tests (based on 1000 permutations). Empirical P-values in such tests, are calculated as the proportion of times that the difference of averages between two categories in a permuted dataset is equal or larger than the observed difference.

## Authors' contributions

TM–B, EEE and AN designed the overall project. TM–B, ZC, XS and AN analyzed the data. TM–B and AN wrote the manuscript.

## Supplementary Material

Additional file 1 Construction of Dataset 2 (Non-overlapping intraspecific dataset). There are the 3 main steps to construct Dataset 2. STEP1, we constructed the "coverage map", basically we recorded the bound coordinates of overlapping SDs. STEP 2, we labeled every SD as belonging to telomeres, centromeres, HSA19, sexual chromosomes, inverted and non-rearranged zones and breakpoints. STEP 3, we kept as a sample of the region in the "coverage map" those SDs that ha d the longer paralogous copy in an equivalently labeled region. Click here for file

Additional file 2 The construction of Dataset 3 (Non-overlapping, interspecific divergence dataset). We split every zone in the coverage map of WGS chimpanzee reads in windows of 5000 bp. For every one of those inner windows, divergence (K_w i) was calculated as the average of divergences of every chimpanzee read against human sequence (B35) (see B). Finally the averages of all windows were joined in a single average divergence of the coverage zone (K total) (see A) Click here for file
