# Supplementary figures and images for: The genomic distribution of intraspecific and interspecific sequence divergence of human segmental duplications relative to human/chimpanzee chromosomal rearrangements
Source: BMC Genomics. 2008 Aug 12;9:384. doi: 10.1186/1471-2164-9-384 (PMC2542386; doi:10.1186/1471-2164-9-384)

## STEP 1

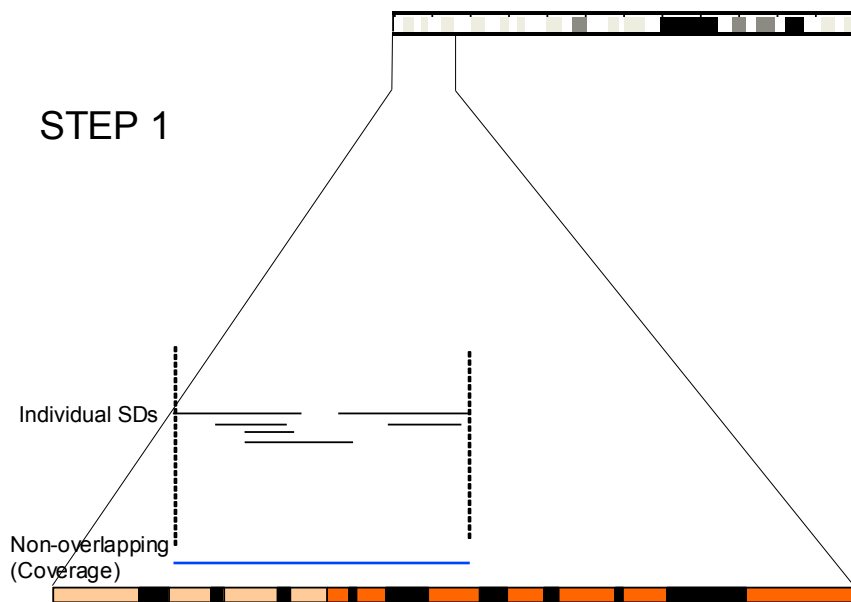

## STEP 2

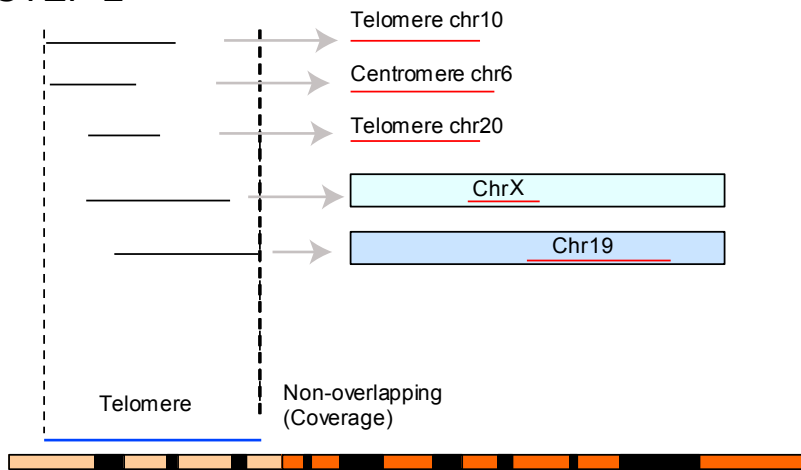

## STEP 3

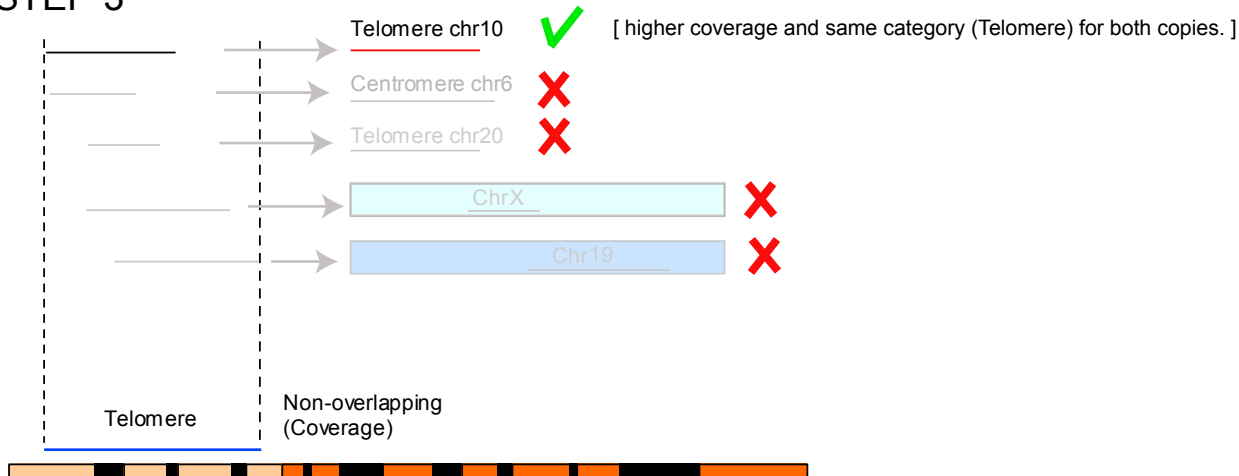

Supplement: Additional file 1 — Construction of Dataset 2 (Non-overlapping intraspecific dataset). There are the 3 main steps to construct Dataset 2. STEP1, we constructed the "coverage map", basically we recorded the bound coordinates of overlapping SDs. STEP 2, we labeled every SD as belonging to telomeres, centromeres, HSA19, sexual chromosomes, inverted and non-rearranged zones and breakpoints. STEP 3, we kept as a sample of the region in the "coverage map" those SDs that ha d the longer paralogous copy in an equivalently labeled region. [file 1471-2164-9-384-S1.pdf]

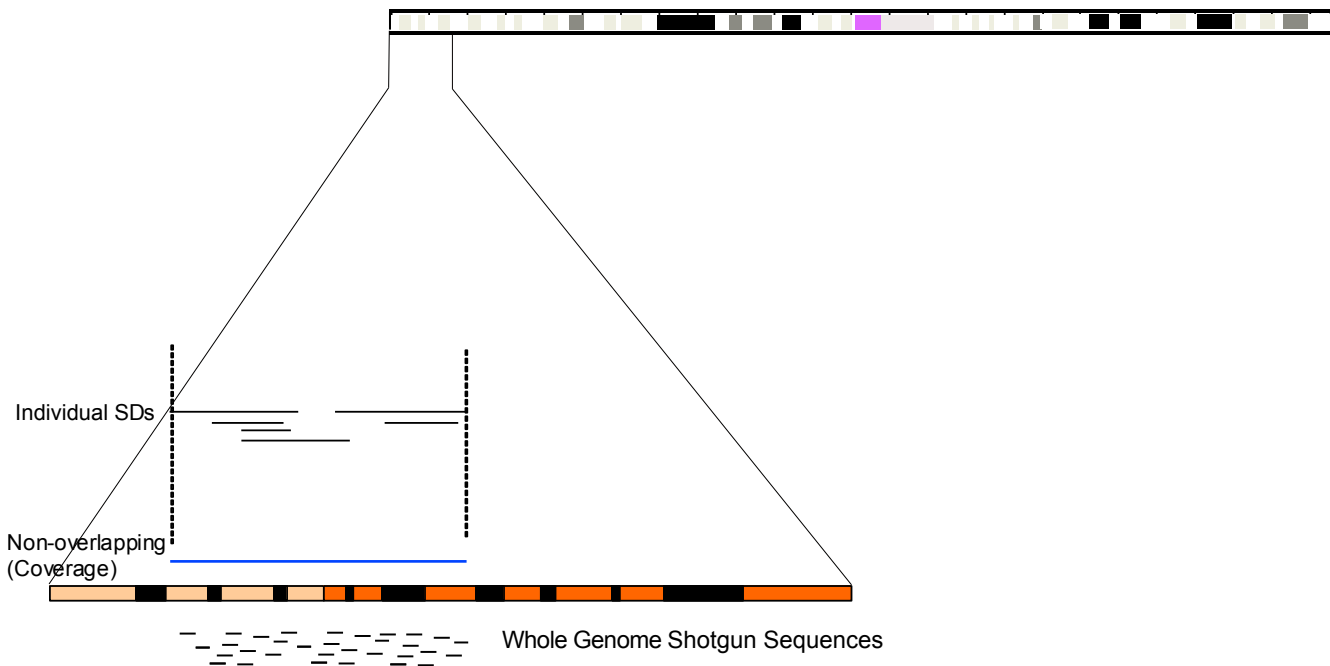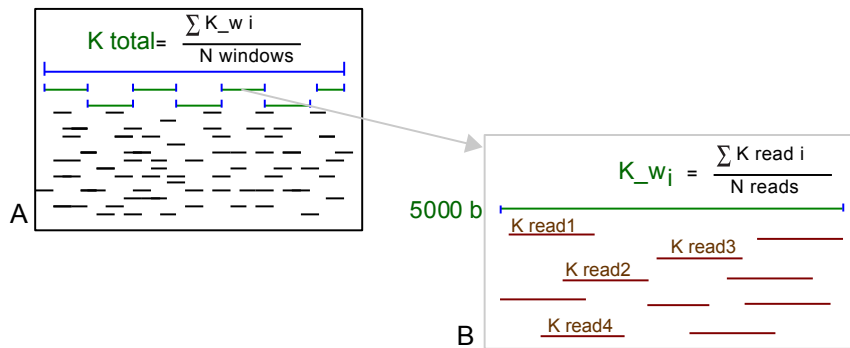

Supplement: Additional file 2 — The construction of Dataset 3 (Non-overlapping, interspecific divergence dataset). We split every zone in the coverage map of WGS chimpanzee reads in windows of 5000 bp. For every one of those inner windows, divergence (K_w i) was calculated as the average of divergences of every chimpanzee read against human sequence (B35) (see B). Finally the averages of all windows were joined in a single average divergence of the coverage zone (K total) (see A) [file 1471-2164-9-384-S2.pdf]
